# Evaluation et analyse de l'insomnie en hémodialyse chronique

**DOI:** 10.11604/pamj.2014.19.221.4444

**Published:** 2014-10-28

**Authors:** Ryme El Harraqui, Naima Abda, Yassamine Bentata, Intissar Haddiya

**Affiliations:** 1 Service de Néphrologie-Hémodialyse, Centre Hospitalier Al Farabi, Oujda-Maroc; 2 Laboratoire de Biostatistiques, Faculté de Médecine et de Pharmacie, Université Mohamed Ier, Oujda-Maroc

**Keywords:** Insomnie, hémodialyse chronique, âge, ancienneté de dialyse, fréquence de dialyse, comorbidités, dépression, Insomnia, chronic hemodialysis, age, length of dialysis, dialysis frequency, comorbidity, depression

## Abstract

L'insomnie est aussi fréquente chez les hémodialysés chroniques qu'elle est négligée par le personnel soignant, davantage attelé à gérer les complications qui accompagnent l'insuffisance rénale chronique terminale et la dialyse chronique. Notre travail a eu pour but de déterminer la prévalence de l'insomnie et les facteurs qui lui sont associés, afin de pouvoir, dans un temps futur, lui dédier une stratégie de prise en charge adaptée. Il s'agit d'une étude transversale incluant 93 hémodialysés chroniques, dont nous avons étudié les données sociodémographiques, biologique, dialytiques; nous avons également collecté les caractéristiques de l'insomnie, et enfin mis en exergue les facteurs associés à sa survenue. L'insomnie a été définie par un score total supérieur ou égal à 15 au questionnaire de l'I.S.I (Insomnia Severity Index). Ainsi, la prévalence de l'insomnie est de 67,7% (n=63); l’âge moyen est de 55,6±12,7 (22-82) ans, le sexe ratio de 24 Hommes/39 Femmes, la durée moyenne en hémodialyse de 94,6 ± 58,2 (6-252) mois. 45 des patients insomniaques sont dialysés deux fois par semaine. Cette insomnie concernait le début de la nuit dans 52,3% des cas, le milieu de la nuit dans 55,5% des cas, et le petit matin dans 19,04% des cas. Les troubles associés à cette insomnie ont été: la somnolence diurne dans 79,3% des cas (n=50), le syndrome de la jambe sans repos dans 17,4% des cas (n=11), et les cauchemars dans 30,1% des cas (n=19). Une prise régulière d'hypnogènes a été notée chez 20 patients (31,7%). En analyse univariée, les facteurs associés à la survenue de l'insomnie sont l’âge avancé des patients, le sexe féminin, l'ancienneté en hémodialyse, la fréquence de deux séances de dialyse par semaine, le taux d'urée en prédialyse, le score de Charlson, la présence d'une douleur chronique (depuis plus de trois mois), la dépression et enfin l'hypertension artérielle. En analyse multivariée, on retient l’âge avancé des patients, le sexe féminin, l'ancienneté en dialyse et la fréquence de deux séances de dialyse par semaine. En conclusion, les troubles du sommeil et en particulier l'insomnie chez les hémodialysés chroniques sont fréquents et multifactoriels. Une attention particulière doit leur être accordée, incluant une collaboration régulière et étroite entre néphrologues et psychologues, psychiatres et spécialistes du sommeil.

## Introduction

Le sommeil est un facteur d’équilibre et de bonne santé encore plus indispensable chez les personnes fragilisées physiquement. Or, comme le montrent les études, c'est dans ce genre de population qu'il est le plus souvent compromis par les atteintes organiques et psychiques associées à la maladie, et les patients en hémodialyse n’échappent pas à cette règle. Cependant, c'est également dans ce genre de population aux tares multiples et aux lourdes comorbidités qu'il a tendance à être négligé au profit de complications plus concrètes et qui engagent de façon plus directe le pronostic vital ou fonctionnel des patients. Dans notre travail, nous nous sommes fixés pour but de déterminer la prévalence et les facteurs associés à l'insomnie au sein de notre population de patients hémodialysés chroniques.

## Méthodes

Notre travail a consisté en une étude transversale monocentrique menée au service d'hémodialyse du Centre Hospitalier Al Farabi d'Oujda en Mars 2012. Nous avons évalué la prévalence de l'insomnie à l'aide d'un questionnaire précis en français et/ou traduit en arabe dialectale, et établi à partir du questionnaire de l'I.SI (Insomnia Severity Index). Ce questionnaire comporte sept questions dont les réponses sont notées de 0 à 4, la somme des notes obtenues formant un score pouvant varier entre 0 et 28. Fidèlement à ce questionnaire, nous avons défini l'insomnie à partir d'un score minimal obtenu de 15 (annexe 1). Nous nous sommes également intéressés: au rythme des insomnies; leurs horaires; leurs explications présumées par les patients.

Les patients dont le score était inférieur ou égal à 14 ont été classés dans un groupe « témoin » ou groupe « sans insomnie ». Nous avons ainsi pu distinguer deux groupes: le groupe « témoin » (groupe « sans insomnie ») et le groupe « insomnie+ », chez lesquels nous avons comparé les paramètres: anthropométriques (âge, sexe, niveau d’éducation); cliniques (néphropathie initiale, tares associées, symptomatologie clinique, indice de masse corporelle IMC, prise médicamenteuse, respect des règles hygiéno-diététiques, niveau d'activité physique); biologiques (numération sanguine, désordres minéralo-osseux, électrolytiques, anomalies cardiovasculaires, bilan thyroïdien); et dialytiques. (Durée en dialyse, rythme et durée des séances, poids sec, prise de poids interdialytique).

L'analyse statistique a été réalisée à l'aide du logiciel SPSS 17.0. Les variables quantitatives sont exprimées en moyennes-écarts-types quand elles répondent à la loi normale et en médianes-extrêmes quand elles sont hors la loi normale. Leur comparaison est réalisée à l'aide du test statistique de Student. Les variables qualitatives sont exprimées en pourcentages. Leur comparaison est réalisée à l'aide du test de Chi deux. La différence est jugée statistiquement significative pour un p< 0,05. Au terme de cette analyse statistique, nous avons pu mettre en exergue les facteurs associés à la survenue de l'insomnie.

## Résultats

Notre population totale comptait 93 hémodialysés chroniques, dont l’âge médian était de 51,5±15,5 (7-83) ans. Les sujets âgés (d’âge supérieur ou égal à 65 ans) représentaient 20,4% de l'ensemble de nos patients (n=19). Le sexe ratio H/F était de 0,978 (46H/47F). L'Indice de Masse Corporelle (IMC) moyen était de 23 ±3,07 (16,78-37) kg/m^2^. On note par ailleurs que 29% des patients (n=27) ont un IMC inférieur à 20 kg/m^2^ SC. L'ancienneté moyenne en hémodialyse était de 82±56 (4-252) mois. Les étiologies de l'insuffisance rénale chronique terminale (IRCT) de ces patients sont résumées dans le Tableau 1. 58 de nos patients avaient une fréquence de 2 séances de 4 à 4,5 heures de dialyse par semaine, tandis que les 35 restants étaient dialysés à raison de 3 fois par semaine pendant une durée de 4 heures par séance. La prise de poids interdialytique moyenne (PPID) était de 3,06± 0,817 (1-4,98) kgs. Le Kt/Vsp moyen était de 1,4 ±0,17 (1,1-2). 32 patients étaient porteurs d'une cardiopathie tout type confondu, objectivée à l’échocardiographie transthoracique, et 22 patients étaient sujets à des épisodes d'hypotension intradialytique (HID).

*NB: L'HID a été définie comme la survenue d'une baisse brutale de la tension artérielle d'au moins 30% par rapport à sa valeur initiale durant la séance d'hémodialyse ou immédiatement après*.

**Tableau 1:** Comparaison des deux groupes « sans insomnie » et « avec insomnie » et analyse statistique des facteurs associés à l'insomnie

Paramètres	Population totale: n=93	Gpe sans Insomnie: n=30	Gpe avec Insomnie: n=63	*P*	*Odds ratio*	*Intervalle de confiance*
Age (ans)	51,5±15,5	40,7 ± 15,8	55,6 ± 12,7	<0,001	1,078	1,038-1,119
Sexe (n)	46H/47F	22H/8F	24H/39F	0,001	4,469	1,718-11,621
Néphropathie initiale (n)				*		
* Indéterminée*	42	14	28	*-*	*-*	*-*
* Diabétique*	12	3	9	*-*	*-*	*-*
* Vasculaire*	11	4	7	*-*	*-*	*-*
* PKR*	15	2	13	*-*	*-*	*-*
* Glomérulaire*	5	2	3	*-*	*-*	*-*
* NTIC*	8	5	3	*-*	*-*	*-*
Ancienneté en HD (mois)	82 ± 56	45,6 ± 32,1	94,6 ± 58,2	<0,001	1,021	1,01-1,032
Patients dialysés deux fois/semaine (n)	58	13	45	0,01	0,306	0,124-0,754
PPID (kgs)	3,06 ±0,817	2,84±0,93	3,17 ± 0,74	*0,061*	1,668	0,969-2,872
Kt/v	1,4 ± 0,17	1,38 ± 0,13	1,41 ± 0,18	*0,312*	4,06	0,272-60,626
Non respect des RHD: n (%)	20 (21,5)	3 (10)	17 (27)	*0,074*	0,301	0,081-1,121
Taux d'urée avant dialyse (g/l)	3,6 ± 0,65	4 ± 0,61	3,5 ± 0,6	0,001	0,269	0,123-0,585
Hémoglobinémie médiane (g/dl)	10,9 ± 2,54	10,25 ± 2,5	11 ± 2,54	*0,179*	1,129	0,946-1,347
PTH 1-84 (pg/ml)	546 ± 424	569,9 ± 420	565 ± 430,2	*0,379*	1	0,999-1,002
Réserve alcaline avant dialyse (méq/l)	21 ± 2,95	23 ± 3,85	21,45 ±2,32	*0.185*	0,833	0,636-1,091
Calcémie médiane (mg/l)	90 ± 8,19	91,5 ± 7,9	88,9 ± 8,26	*0,887*	1,004	0,952-1,059
Phosphatémie médiane (mg/l)	40 ± 21,1	49,9 ± 22,6	41,7 ± 20,1	*0,624*	1,005	0,984-1,027
Indice de masse corporelle (kg/m^2^)	23 ± 3,07	23 ± 2,91	23,1 ± 3,17	*0,852*	1,014	0,876-1,174
Pré- albuminémie médiane (g/l)	0,27±0,11	0,27 ± 0,11	0,3 ± 0,11	*0,654*	0,424	0,01-18,06
Albuminémie médiane (g/l)	31,5 ± 10,4	30,5 ± 10	33,5 ± 10,5	*0,394*	1,019	0,976-1,063
Taux de CRP médiane (g/l)	4,5 [0-40]	9,13 ± 9,9	6,2 ± 7,23	*0,791*	0,993	0,943-1,046
Score de Charlson (n)	4± 2,2[2-14]	3,5 ± 1,52	4,85 ± 2,4	0,006	1,467	1,103-1,951
Patients avec DL chronique >3 mois: n (%)	63 (67,7)	13 (43,3)	50 (79,3)	0,012	3,365	1,312-8,63
Patients dépressifs	17 (18,2)	6 (20)	11 (31,3)	*0,5*	0,695	0,239-2,021
NSE bas: n (%)	64 (68,8)	20 (66,6)	44 (69,8)	*0,757*	1,158	0,457-2,936
Patients analphabètes: n (%)	61 (65,5)	19 (63,3)	42 (66,6)	*0,752*	1,158	0,467-2,873
Patients hypertendus: n (%)	57 (61,3)	14 (46,6)	43 (68,2)	0,048	2,457	1,007-5,996
Patients porteurs de cardiopathie: n (%)	32 (34,4)	9 (30)	23 (36,5)	*0,537*	1,342	0,527-3,415
Comorbidités associées à l'IRCT: n				*		
*Diabète*	13	4	9	*-*	-	-
* Insuffisance cardiaque congestive*	6	1	5	*-*	-	-
* Antécédent d'IDM*	2	0	2	*-*	-	-
* Antécédent d'AVC*	1	0	1	*-*	-	-
* Hémiplégie*	1	0	1	*-*	-	-
* Tumeur solide avec métastase*	1	0	1	*-*	-	-
* Tumeur solide sans métastase*	1	0	1	*-*	-	-
* Pathologie hépatique modérée à sévère*	3	1	2	*-*	-	-

PKR: Polykystose rénale; NTIC: Néphrite tubulo-interstitielle chronique; NGC: Néphropathie glomérulaire chronique; IDM: infarctus du myocarde; AVC: accident vasculaire cérébral; RHD: règles hygiéno-diététiques.

* Test statistique invalide (insuffisance des échantillons)

Le score de Charlson moyen était de 4 ± 2,2 (2-14). La liste des comorbidités enregistrées est résumée dans le Tableau 1. Le Tableau 1 résume l'ensemble des paramètres biologiques évalués.

63 patients (67,7%), souffraient d'insomnie: du début de la nuit dans 52,3% des cas; du milieu de la nuit dans 55,5% des cas; et du petit matin dans 19,04% des cas. Parmi ces patients: 24 étaient dialysés le matin; 24 l'après-midi; et 15 en alternance matin et après-midi dans la même semaine; 45 étaient dialysés deux fois par semaine; et 18 trois fois par semaine. Leur sexe ratio était de 24H/39F. Leur âge moyen était de 55,6±12,7 (22-82) ans. Leur durée moyenne en hémodialyse était de 94,6 ± 58,2 mois. (6-252) mois.

Les troubles associés à cette insomnie ont été: la somnolence diurne dans 79,3% des cas (n=50); le syndrome de la jambe sans repos dans 17,4% des cas (n=11); et les cauchemars dans 30,1% des cas (n=19). Une prise régulière d'hypnogènes a été notée chez 20 patients (31,7%).

En analyse statistique univariée, les facteurs corrélés à la survenue de l'insomnie sont: l’âge avancé des patients, le sexe féminin, l'ancienneté en hémodialyse, la fréquence de deux séances de dialyse par semaine, le taux d'urée en prédialyse, le score de Charlson, la présence d'une douleur chronique (depuis plus de trois mois), la dépression, et enfin l'hypertension artérielle.

En analyse statistique multivariée, les facteurs statistiquement associés à l'insomnie sont: l’âge avancé des patients, le sexe féminin, l'ancienneté en dialyse, et la fréquence de deux séances de dialyse par semaine.

Les causes attribuées aux insomnies sont: l'anxiété dans 37,3% des cas (n=25), la dépression dans 17,4% des cas (n=11), et les douleurs chroniques nocturnes chez 79,3% des patients (n=50), d'origine ostéo-articulaire, digestive et neurologique chez respectivement 41, 4 et 5 patients.

Parmi les 46 patients souffrant de troubles anxiodépressifs, 32 sont des femmes (69,5%). Enfin, en analyse de corrélation, on constate que les patients dont les valeurs de PTH1-84 se situent dans la fourchette fixée par les recommandations des K-DOQI ont globalement moins de risque de développer une insomnie par rapport aux autres patients dont les valeurs de PTH sont en deçà ou au-dessus des valeurs recommandées, même si cette constatation n'est pas statistiquement significative (Tableau 2).

**Tableau 2:** Analyse de corrélation selon les valeurs de PTH1-84

PTH1-84 (pg/ml)	*p*	Odds ratio
<150	0,463	1,625
150-450	0,514	1,333
>450	0,231	1,9

## Discussion

Il est de plus en plus clairement établi que les troubles du sommeil, en particulier l'insomnie, le syndrome d'apnées du sommeil et le syndrome des jambes sans repos, sont très fréquents chez les patients atteints d'insuffisance rénale chronique, qu'ils soient dialysés ou non [1, 2]. Il a été également rapporté que ces troubles sont associés à un risque plus élevé de morbi-mortalité [3]. A titre d'exemple, Elder et ses collaborateurs ont démontré dans une large étude portant sur 11351 patients répartis dans 308 centres de dialyse, que le risque de morbi-mortalité était plus élevé chez les patients souffrant de troubles du sommeil [4]. Enfin, l’équipe de Montpellier a établi une relation entre les troubles du sommeil et le risque cardiovasculaire chez 36 patients hémodialysés chroniques [5].

De plus, l'influence néfaste de ces troubles sur la qualité de vie des patients a également été prouvée par différents auteurs [6–8]. C'est le cas par exemple de Théofilou, qui a mené une étude auprès de 144 patients répartis dans trois centres de dialyse différents, parmi lesquels 84 hémodialysés chroniques et 60 patients en dialyse péritonéale continue ambulatoire. Au terme de celle-ci, il a conclu que l'insomnie et les symptômes qui lui sont associés était responsable d'une altération de la qualité de vie des patients et cela quelque soit la modalité de dialyse à laquelle ils adhèrent. C'est dire la nécessité de porter de l'intérêt à ces troubles, l'importance de les rechercher et les expliquer afin de pouvoir les prévenir. Or, il s'avère que jusqu’à cette dernière décennie, cet aspect de la prise en charge des patients dialysés ait paradoxalement été négligé, tant les complications de l'insuffisance rénale chronique, les lourdes comorbidités qui peuvent s'y associer, ainsi que la maitrise des paramètres physicochimiques de dialyse étaient au centre des préoccupations et au cœur des débats.

Quoiqu'il en soit, tous les auteurs s'accordent à dire actuellement que les désordres du sommeil ont une prévalence élevée en dialyse, avec souvent des taux comparables entre hémodialyse et dialyse péritonéale [9] et notre population ne fait pas exception à la règle, puisque nous retenons que 67,7% de nos patients, c'est-à-dire que deux patients sur trois admettent en souffrir. Les causes de l'insomnie et des troubles qui lui sont souvent associés sont multifactorielles et sont liées à la maladie rénale elle-même, aux traitements ainsi qu’à des facteurs psychosociaux.

Chez les hémodialysés chroniques, des facteurs supplémentaires ont été incriminés dans la survenue de l'insomnie. Ainsi, dans une étude menée sur 69 patients, une durée en hémodialyse supérieure à un an était statistiquement associée à la survenue de l'insomnie [10]. En revanche, dans cette même étude, les patients dialysés depuis moins d'un an étaient moins enclins à la dépression, ce qui est surprenant étant donné qu'insomnie et dépression sont souvent associées en hémodialyse, comme c'est le cas pour Kao notamment [11]. Knezevigh s'est en revanche intéressé à l'impact des modalités d'hémodialyse sur la survenue et la sévérité de l'insomnie à travers une étude menée sur 122 patients répartis entre hémodialyse avec membrane high flux, low flux et hémodiafiltration [12]. Son analyse statistique révèle que la sévérité de l'insomnie jugée selon le score ISI (Insomnia severity index) est plus marquée chez les patients en hémodialyse low flux que chez ceux en hémodialyse high flux. Par contre, il n'y avait pas de différence entre les patients sous hémodialyse high flux et les patients sous hémodiafiltration. Certains ont démontré la relation entre l'inflammation et l'insomnie [7, 8, 13]. Par exemple, Razeghi a démontré, au travers d'une étude menée sur 108 hémodialysés chroniques, qu'un taux de CRP supérieur à 3,8 µg/ml était corrélé avec un p statistiquement significatif à la survenue de l'insomnie. D'autres retiennent l'anémie comme facteur associé à l'insomnie [7]. Parfois encore, le niveau bas d'activité physique et/ou sportive a été statistiquement associé de façon significative à l'insomnie [14]. Par ailleurs, certains auteurs se sont intéressés au rythme saisonnier des troubles du sommeil en hémodialyse [15].

D'autres facteurs de risque ont également été fréquemment incriminés, tels que: l'horaire de dialyse, la qualité de dialyse, l'urémie [16], la malutrition [7, 8, 16], la calcémie, ou encore la phosphatémie. Enfin, certains auteurs n'associent l'insomnie à aucun facteur de risque particulier [17]. Dans notre travail, les facteurs de survenue des troubles du sommeil retenus dans notre population ont été l’âge, le sexe féminin, l'ancienneté en dialyse, le taux d'urée avant dialyse et la fréquence de deux séances de dialyse par semaine. On notera par ailleurs que l'augmentation du taux de PTH est parallèle à celle du risque d'insomnie: c'est une tendance remarquée par notre analyse statistique même s'il n'y a pas de significativité.

## Conclusion

Les troubles du sommeil nuisent à la qualité de vie des patients de dialyse et de ce fait à leur combativité face à la maladie. S'ils peuvent sembler anodins à coté des différents désordres osseux, cardiaques ou autres, ils peuvent néanmoins entraver la bonne adhésion des hémodialysés à leur prise en charge. Une attention particulière devrait donc leur être accordée, incluant une collaboration régulière et étroite entre néphrologues et psychologues, psychiatres et spécialistes du sommeil.
